# Effect of different conditioning methods of traditional Chinese health exercise on lung function in healthy middle-aged and elderly people: study protocol for a randomized controlled trial

**DOI:** 10.1186/s13063-021-05980-5

**Published:** 2022-01-03

**Authors:** Wenlong Li, Yapei Song, Qiuping Xiang, Xinlei Wang, Xiaoyun Wei, Tonggang Fan

**Affiliations:** 1 College of Chinese Wushu, Shanghai University of Sport, 200 Hengren Road, Yangpu District, Shanghai, China; 2 Office of P.E teachers, Shanghai Institute of Tourism, Shanghai, China; 3 Yueyang Hospital of Integrated Traditional Chinese and Western Medicine, Shanghai University of Traditional Chinese Medicine, Shanghai, China

**Keywords:** Exercise therapy, Forced vital capacity, Lung function, Traditional Chinese health exercise

## Abstract

**Background:**

Lung function is highly age-dependent as it decreases in varying degrees with age, even in healthy people. Decreased lung function results in less elastic lung tissue, reduced chest wall compliance, reduced area for gas exchange, and even a variety of chronic diseases. Traditional Chinese health exercise (TCHE) has three components: “breath regulation,” “body regulation,” and “heart regulation,” which play an important role in the improvement of lung function. However, which component has the most significant effect on lung functioning remains unclear. Therefore, depending on the modality of conditioning, TCHEs will be divided into three exercise intervention groups: breath regulation group, body regulation group, and heart regulation group, in order to explore the magnitude of the effect of the different modalities of conditioning on the improvement of lung function.

**Methods:**

The prospective, parallel, single-blind, randomized controlled trial will evaluate the effects of different conditioning methods of TCHE on lung function in middle-aged and elderly people. The study subjects are healthy middle-aged and elderly adults, who will be randomly divided into the “breath regulation group,” “body regulation group,” “heart regulation group,” and “control group.” The control group will receive health education. Health education and exercise intervention in the three intervention groups will be provided for 6 months, 5 times a week, with each session lasting 60 min. The outcomes of interest include changes in the pulmonary function tests measured at baseline and 3 and 6 months after the beginning of the intervention. The primary outcome is the forced vital capacity (FVC), while the secondary outcomes include forced expiratory volume in 1 s (FEV1), FVC/FEV1 ratio, vital capacity (VC), and maximal voluntary ventilation (MVV).

**Discussion:**

This study will assess the effects of different conditioning methods of TCHE on lung function in middle-aged and elderly people. The final findings of this study will validate the effectiveness and safety of TCHE on lung function interventions in middle-aged and elderly people.

**Trial registration:**

China Clinical Trial Registry ChiCTR2100052687. Registered on November 3, 2021

**Supplementary Information:**

The online version contains supplementary material available at 10.1186/s13063-021-05980-5.

## Background

Pulmonary function is highly age-dependent, with a slow decline starting at 20–30 years of age [1]. Decreased lung function results in the loss of lung tissue elasticity, reduced chest wall compliance, and reduced area for gas exchange [2]. Even in healthy people, lung function decreases with age [3]. When lung function declines too rapidly, the incidence of related diseases, such as respiratory and cardiovascular diseases, greatly increases [4]. Chronic obstructive pulmonary disease (COPD) is one of the most common diseases presenting with decreased pulmonary function, and the Global Burden of Disease Study 2015 estimated that approximately 174 million people suffer from COPD worldwide [5]. The rapid decline in lung function is also a significant predictor of death [6]. In 2015, COPD was ranked the third cause of age-standardized mortality in men and women globally, with approximately 3.2 million patients dying of the disease [7]. Middle-aged and older people with lower lung function have a faster rate of decline in cognitive abilities, such as memory and executive functions, than those with higher lung function [8]. Research shows that early exercise intervention and care for healthy older people can effectively reduce the incidence of chronic diseases and reduce health care costs [9]. Due to accelerated aging, the population of people aged > 65 in China was projected to reach 400 million by 2050, of which 150 million were above the age of 80 [10]. Therefore, it is very important to intervene in the early pulmonary function of middle-aged and elderly subjects.

Exercise greatly affects health status, and a lack of adequate exercise may cause systemic inflammation, cardiac dysfunction, and decreased lung function leading to most chronic diseases [11–13]. For most healthy adults, the benefits of exercise far outweigh the risks, and regular exercise is beneficial to improve and maintain the health of lung function [14]. For people with respiratory diseases, exercise training is regarded as the cornerstone of lung rehabilitation since it promotes disease rehabilitation, improves postoperative recovery, and prevents complications [13, 15]. Therefore, regular exercise is of great significance for both healthy individuals and those with respiratory diseases. However, the importance of exercise remains overlooked by some doctors and patients [16].

Traditional Chinese health exercise (TCHE) is an effective means of maintaining health and fitness and managing stress [17]. It regulates and enhances the functions of various parts of the human body through constant postural changes, breathing exercises, and mindfulness. It also presents characteristics of traditional Chinese medicine, including the application of meridians and acupoints and the regulation of qi, which increases its medical value [18]. TCHE plays important roles in the improvement and treatment of lung function, since these methods emphasize a close relationship between exercise and breathing. A number of studies have shown a significant improvement in the lung function of those who practice the TCHE [19–21]. Traditional lung rehabilitation therapy utilizes either physical or breathing exercise only. TCHEs combine these two types of exercise with mindfulness activities, which may have additional beneficial effects on lung function [22]. Different from and in contrast with traditional lung rehabilitation, which focuses on the rehabilitation treatment of patients after illness [15], TCHE focuses on disease prevention, a characteristic that reflects the preventive role of traditional Chinese medicine [18]. All TCHEs (such as Taijiquan, Baduanjin) include three types of regulation, namely “breath regulation,” “body regulation,” and “heart regulation,” all of which are indispensable and present in varying proportions across TCHE. Although previous studies have investigated the effects of TCHEs on lung function, no studies have explored the effects of each of these conditioning modalities on lung function separately. Therefore, depending on the modality of conditioning, the TCHEs will be divided into three exercise intervention groups: breath regulation group, body regulation group, and heart regulation group, in order to explore the magnitude of their effect on the improvement of lung function.

The purpose of this study is to analyze the effects of “breath regulation,” “body regulation,” and “heart regulation” on the improvement of lung function and to explore which modality is the most effective intervention for healthy middle-aged and older subjects.

## Methods

### Trial design

The prospective, parallel, single-blind, randomized controlled trial will evaluate the effects of different conditioning methods of TCHE on lung function in middle-aged and elderly people. It fulfills the requirements of the Standard Protocol Items: Recommendations for Interventional Trials (SPIRIT) checklist [23].

**Fig. 1:**
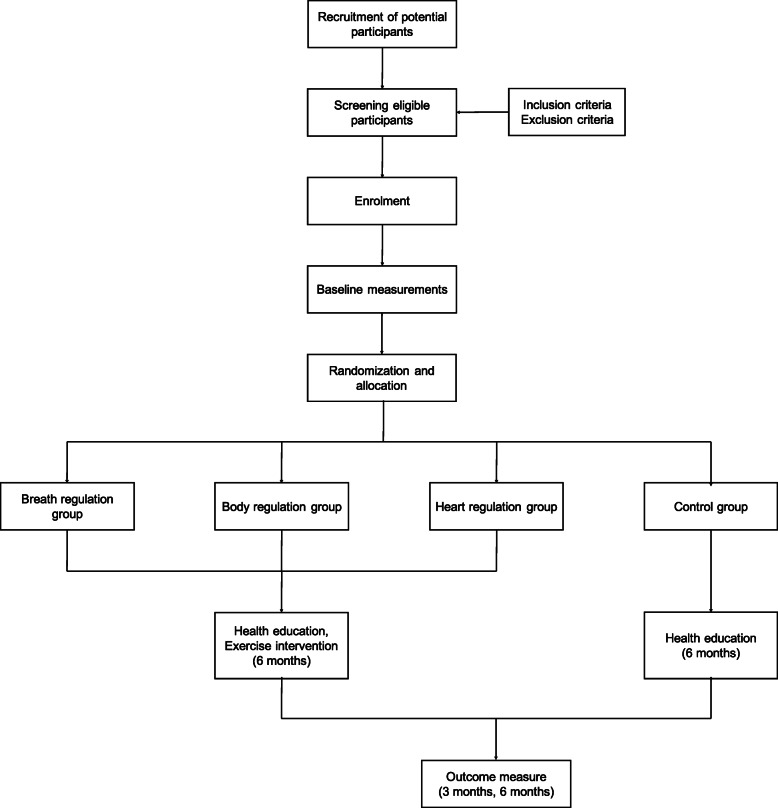
Flow chart of the research scheme

**Fig. 2:**
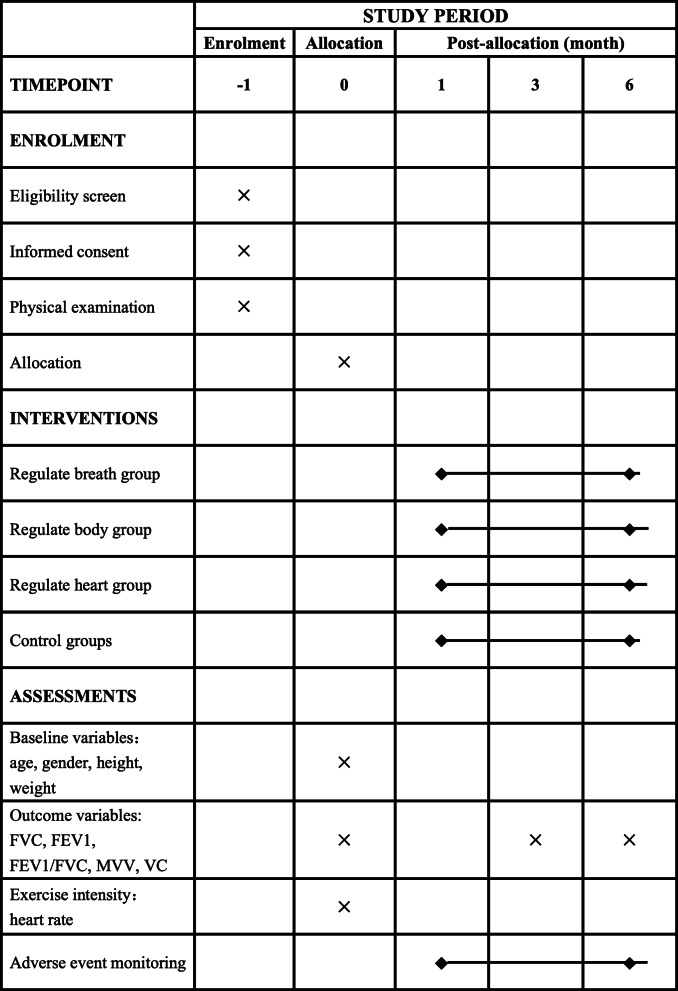
SPIRIT figure. SPIRIT figure showing the times points for enrollment, interventions, and assessments. FVC, forced vital capacity; FEV1, forced expiratory volume in 1 s; MVV, maximal voluntary ventilation; VC, vital capacity

The subjects will be recruited from Mengcheng County, Anhui Province, China. A simple randomization will be used to divide them into a breath regulation group, body regulation group, heart regulation group, and control group in a 1:1:1:1 proportion. The subjects in the control group will receive health education only and maintain their previous lifestyle, and the three experimental groups will undergo exercise intervention (including warm-up, relaxation, and stretching) and health education for 6 months, 5 times a week, with each session lasting 60 min. The main and secondary results will be tested at baseline and at 3 and 6 months after the beginning of the intervention. An overview of the research program flow is presented in Fig. 1. The SPIRIT flow chart of the time points for enrolment, interventions, and assessments is shown in Fig. 2.

### Eligibility criteria

To facilitate the recruitment of subjects, exhibition stands will be set up at the Mengcheng Museum, Mengdie Square, Zhuangzi Community, and at the Second People’s Hospital of Mengcheng County, Anhui Province, and leaflets will be handed out. The conditions of each potential participant will be assessed through on-site enquiries and telephone interviews. Men and women who meet the following inclusion criteria will be eligible: (1) aged 45–75 years; (2) no history of respiratory diseases; (3) no previous practice of systematic TCHE, such as Taijiquan and Baduanjin; and (4) signed written agreement to participate voluntarily after fully understanding the research purpose and procedures. The exclusion criteria are as follows: (1) limited physical activity or other factors that hinder the performance of the exercise intervention, (2) current practice of other regular exercise regimens, (3) cognitive impairment, and (4) participation in other clinical studies.

### Sample size calculation

Considering the results of a previous study that used forced vital capacity (FVC) as the primary outcome index [24], the sample size was calculated by one-way ANOVA in Power Analysis and Sample Size version 15.0.5 (PASS 15.0.5 NCSS, LLC, USA). With a power of 80%, an alpha of 0.05, a standard deviation of 48, a standard deviation of the means of 8.16, and a dropout rate of 20%, we aim to recruit a minimum of 120 subjects per each of the four groups.

### Randomization and blinding

After screening, interview, and evaluation of qualifications, the subjects who meet the inclusion criteria will be randomly divided into the breath regulation group, body regulation group, heart regulation group, and control group using a random number generator. The random list will be generated by an independent person using a computer to ensure the anonymity of the allocation.

The distribution order will be saved in sequential numbers in opaque and sealed-bound envelopes with aluminum foil inside to ensure that the envelopes are not visible in bright light. After the details of the subjects are written on the envelope, the envelope can be opened.

The lead investigator and data analyst will not participate in any intervention. The blind method will be used for the outcome assessor and data analyst to prevent the selective reporting of outcome variables. The subjects, coaches, researchers, and follow-up personnel will not be blinded, due to the particularity of sports intervention. The evaluators will not participate in the recruitment and distribution of subjects, and subjects, coaches, and researchers will be asked to refrain from discussing the intervention measures with the evaluators before the end of the experiment. In the event of serious side effects, blinding can be removed at the discretion of the outcome assessor.

### Interventions

In addition to health education, the subjects in the three intervention groups (“breath regulation group,” “body regulation group,” and “heart regulation group”) will perform the exercise intervention 5 times a week for 60 min (including a 10-min warm-up exercise, a 40-min stretching exercise, and a 10-min relaxation) for 6 months. The control group will not carry out any interventions except health education. All subjects will be asked to maintain their pre-intervention lifestyle and refrain from any strenuous exercise that may affect the test results. Air quality may be a factor in the effectiveness of the intervention [25]; therefore, the intervention groups will receive exercise intervention from 7:00 to 8:00 in the morning in different parks in Mengcheng County (different groups in different locations). Borg ratings of perceived exertion (RPE) (1982) [26] are a practical, simple, and cheap method to monitor internal load [27]. This scale has a high correlation with heart rate, exercise load, fat consumption, sugar consumption, and total energy output, and can be used as an effective index to judge load intensity [28]. All participants in the three intervention groups will wear a heart rate meter (polar s610i, Finland) to measure their heart rate during each exercise. Subjects will receive an education session on the RPE scale prior to the start of the intervention so that the RPE can be assessed at any time during the exercise intervention. Before the experiment, training courses will be conducted with the help of the staff, including coaches and result evaluators. All the coaches have more than 10 years of experience in TCHE and will undergo one-to-one training before the teaching sessions. The exercise contents of the three groups were selected from restored ancient health books approved by experts in fitness Qigong, traditional Chinese medicine, and related fields [29–32].

#### Breath regulation group

In the breath regulation group, the subjects will perform eight movements to practice breathing while moving to the tune of assigned music. The breathing modes, including natural breathing, nasal suction, mouth spitting, and abdominal and non-abdominal breathing, are simultaneously combined with limb movements, such as flexion and extension of the upper limbs, pulling of the upper limbs, and left and right rotation of the body. The combination of multiple breathing modes may improve respiratory muscle strength and endurance by reducing the relative proportion of the anatomic invalid space of the respiratory tract, enhancing the gas exchange between the alveoli and capillaries, and increasing the range of motion of the diaphragm [33]. Simultaneously, the upper limb movement increases the depth of breathing by stretching and massaging both sides of the body. This dredges the meridians and airways to improve breathing thereby improving lung function.

#### Body regulation group

The eight movements in the body regulation group are performed in a standing position. In contrast with the breathing combined with limb flexion and extension exercise in the breath regulation group, the subjects in the body regulation group need to pay more attention to the strength exercise of the lower limbs while flexing and extending the upper limbs, particularly in the semi-squat position, standing-to-squatting conversion, and one-leg support position. Through flexion and extension and the continuous change of the body’s center of gravity, the muscle tissue is contracted and stretched, dredging the meridians and collaterals. This enhances the strength of the quadriceps femoris and the balance of the body [20]. It is important to note that the strength of the quadriceps femoris is related to lung function [34]. Concurrently, upper limb movements include shoulder and chest expansion, which are conducive to breathing.

#### Heart regulation group

Heart regulation refers to the regulation of the spirit and consciousness, through the modulation of thinking [35]. The twelve movements of the heart regulation group are performed in the standing posture, according to Zhuangzi’s health fables. Each movement corresponds to a health fable. Health fables express the importance of health through metaphorical stories. Zhuangzi is an ancient Chinese figure representative of health preservation, and the term “health preservation” is first found in *Zhuangzi*. The fables regarding health and life philosophy, recorded in *Zhuangzi*, can be combined with body movements to allow better regulation of the spirit and consciousness.

Before practice, the coach explains the implication of each action. During the practice, through a combination of allegory and music, the subjects can keep calm, eliminate distractions, and relax. In mindfulness meditation, the mind focuses on breathing and related acupoints; in exercise meditation, the mind focuses on the coordination of body movement and breathing to guide the movements [36]. Thus, it can regulate the viscera, dredge Qi and blood, and improve lung function.

#### Control group

The control group will receive health education for the prevention and control of middle-aged and elderly chronic diseases without any exercise intervention. Concomitantly, the exercise time and physical labor during the intervention period will be recorded for each subject in the control group.

### Strategies to improve adherence to interventions

Subjects will not be able to change their group during the intervention. To better monitor the subject’s skill practice, all exercises will be conducted under the guidance of a coach in the designated venue until the end of the 6-month intervention. The coach will monitor the attendance before each exercise, and the researchers will conduct telephone follow-ups. In the first 1–2 weeks, the main task will be to learn the movements with the explanation and demonstration of the coaches. Between weeks 3 and 6, the main task will be to improve the standardization of the movements. The coaches will further correct the movements based on the subjects’ basic mastery of the movement structure and strengthen the interaction between different movements. Between weeks 7 and 24, the main task will be to practice repeatedly to increase the subject’s understanding of the action.

### Outcomes

The changes of pulmonary function from baseline to the end of the interventions will be assessed using the pulmonary function evaluation system (MasterScreen, Germany). The main outcome measure is forced vital capacity (FVC), and the secondary outcome measures are the forced expiratory volume in 1 s (FEV1), FEV1/FVC ratio, vital capacity (VC), and maximal voluntary ventilation (MVV). All results will be evaluated at baseline and 3 months and 6 months after the intervention by the same evaluator. The test will be conducted in a room with good ventilation and constant temperature and air pressure to minimize the influence of confounding variables on lung function [37].

The pulmonary function of the patients will be evaluated according to the standards of the American Thoracic Association/European Respiratory Association (ATS/ERS) [38]. Technicians will be required to skillfully grasp the correct operation of the inspection items and have a good service attitude. In the process of inspection, technicians will verbally and non-verbally encourage participants to complete the inspection. Before the test, the technician will explain the purpose and method of the lung measurement examination to the participants. In order for the participants to understand the task of completing maximal inhalation and exhalation, the technicians will demonstrate the proper technique. The height, weight, name, age, and other parameters of the subjects will be recorded. For the measurements of height and weight, subjects will be asked to be barefoot and use light clothes. The subjects will be standing, and the height of the instrument will be adjusted to maintain the head in a front-facing position. During the test, a mouthpiece will be wrapped tightly with the lips, and the nose will be held with both hands to avoid air leakage. Next, the technicians will instruct the participants to inhale or exhale and to avoid coughing or repetitive breathing throughout the exhalation or inhalation without interruption. The test will be conducted from 9 to 11 am to avoid the changes in individual lung function that occur throughout the day.

#### Primary outcome measurements

FVC, which refers to the maximum volume of gas exhaled after inhaling the maximal amount of gas possible, is the primary outcome indicator for this study [39]. It is an important indicator of the presence or absence of resistance in the airways. After breathing calmly, the subject will be requested to inhale to the total lung volume and quickly exhale all the air at once with maximum force. Each subject will repeat the exercise 3 to 8 times until three acceptable and reliable pulmonary function values are obtained. If such values are not obtained after 8 times, we will ask the subject to come for testing the next day so as to avoid inaccurate values due to excessive repetition. For FVC, the acceptable difference between the maximum and the second maximum is within 150 mL [40].

#### Secondary outcome measurements

Secondary outcome indicators in this study include FEV1, FEV1/FVC, MVV, and VC. FEV1 refers to the volume of gas exhaled by forced exhalation in 1 s after maximal inspiration; it is one of the most commonly used parameters to evaluate the degree of lung function damage [41]. The FEV1% predicted value is an important indicator of the degree of lung function impairment. For the FEV1 measurement, the subject will be asked to exhale rapidly within 1 s after maximum inspiration (total lung volume). As with FVC, the difference between the two FEV1 tests should be less than 150 mL.

FEV1/FVC is the most commonly used parameter to evaluate the presence of expiratory airflow obstruction. The Major Respiratory Society guidelines recommend the diagnosis of airflow obstruction when the ratio of FEV1 to FVC is less than a fixed threshold of 0.70 [42]. FEV1/FVC is negatively correlated with age, with lower FEV1/FVC values as age advances [43].

VC is the maximum volume that can be exhaled slowly and deeply after maximal inspiration and is also known as slow vital capacity. It is one of the most commonly used indices of lung capacity [44]. Subjects will be asked to exhale and inhale as deeply as possible evenly at a moderate speed and force. There must be no leakage or repetition of breaths during a cycle of exhalation and inhalation. The VC test is repeated a maximum of 5 times with a difference of less than 150 mL between the best and second-best values.

MVV is the ventilation volume obtained by repeating the maximum spontaneous breathing as fast and as deep as possible in a period of time and is a reliable index for the comprehensive evaluation of the pulmonary ventilation reserve [39, 45]. It is related to the strength of the respiratory muscles, the elasticity of the thorax and lung tissue, and airway resistance. Subjects will be asked to follow the physician’s instructions, after 4–5 normal breaths until the baseline expiratory volume is stable and to keep breathing at maximum respiratory amplitude and fastest respiratory rate for 12–15 s with a 3- to 5-min break between each of the three tests. There should be no air leakage during breathing, and the difference between the best two results should be < 8%.

### Statistical methods

The SPSS statistics software (IBM SPSS STATISTICS, 25.0 version, Armonk, NY) will be used for data analysis and statistical testing. The Kolmogorov-Smirnov test will be used to check whether the data conform to the normal distribution. The average ± standard deviation (SD) will be used to describe continuous variables, and categorical variables will be expressed as *n* (%). Paired sample *t*-test will be used for intra-group comparison, and one-way analysis of variance (ANOVA) and covariance analysis will be used for inter-group comparison. The homogeneity of variance will be tested before ANOVA followed by a comparison of between-group variability using the least significant difference method corrected for test level *α* using Bonferroni adjustment.

Covariance analysis will be used to control other non-interventional factors. Repeated measurement analysis of variance will be used to analyze the intervention effect at different intervention times. The statistical difference may be significant (if *P* < 0.05) and very significant (*P* < 0.01).

All missing data will be analyzed using intention-to-treat analysis. Multiple imputation will be used to fill missing completely at random and missing at random, and last-observation-carried-forward will be used to fill miss not at random data.

### Safety

As the intervention is a simple exercise and not an untested drug or medical device, we do not expect any adverse events to occur. During each exercise, the subject’s heart rate will be monitored; when the heart rate reaches 80% of the heart rate reserve, the exercise will be stopped. After each training session, the subjects will complete the RPE scale, and the exercise intensity will be comprehensively controlled using the heart rate and RPE scale. Adverse effects such as dizziness, fainting, dyspnea, falls, or loss of consciousness during the intervention will be recorded and evaluated by the researchers to determine the relationship between the adverse event and the exercise program. We will use non-systematic approaches to collect the occurrence of adverse events. Subjects will also be encouraged to report any physical discomfort that they might experience during the exercise intervention. All adverse events will be recorded. If a direct injury occurs in connection with this study, the investigator will take appropriate medical treatment and provide basic rehabilitation treatment. In the event of a serious adverse event, the program will be terminated immediately; the investigators will record the event and report it in a trial application to the ethics committee within 24 h.

An oversight committee including a steering committee and a data monitoring committee will be formed. The monitors will include a statistician, a clinical trial scientist, and a physician with a medical background. The statistician will verify and validate the trial data, and the clinical trial scientist will monitor the progress of the trial on a regular basis so that the clinical trial is conducted in strict accordance with the trial protocol. The physician will regularly evaluate the safety and efficacy of the clinical trial progress to ensure that the rights of the subjects in the clinical trial are effectively protected. Moreover, auditors, who are completely independent of the clinical trials, will conduct monthly audits of the raw data, interventions, and clinical trials. If protocol modifications are required during the study, the modified protocol will be submitted to the Ethics Committee of the Shanghai Sports Institute for approval before implementation. Furthermore, no subgroup analysis with mid-term analysis is planned for this study.

No consent is required for the collection of participant information with biospecimens as we will not collect any biospecimens and do not intend to use the subjects’ data in future studies.

### Data management and dissemination

Data will be recorded in pairs, outliers will be identified to avoid entry errors, and auditors will conduct regular spot checks to ensure the authenticity and accuracy of the data.

The data in this experiment, including subject identity and personal information, outcome indicator data, and other related data, will be kept strictly confidential. A list of the participants’ contact information and their corresponding ID numbers will be kept separately from the other data. All paper copy forms will be stored in locked filing cabinets during the test. At the end of the experiment, these files will be scanned and transferred to a password-protected computer. Only authorized research assistants will have access to the final trial dataset.

Upon completion of the trial, the Shanghai Sports Institute Clinical Research Service will be invited to report on the results. The results of the trial will be published in peer-reviewed journals and shared at relevant national and international conferences.

## Discussion

Physical exercise is an important factor that improves the health status of people of all ages [46], and lack of physical exercise is a leading cause of mortality worldwide [47]. Sports practice provides a wide range of health benefits, including the prevention of chronic illnesses and complications and improvement of postoperative recovery speed [13]; no other single intervention or treatment can produce such a wide range of benefits to health [48]. However, not all diseases can be effectively improved by exercise. Different types of diseases require different types of exercise for targeted interventions [13]. The types of exercise recommended in the lung rehabilitation guidelines include endurance, intermittent resistance, and flexibility training [15]. Endurance training is the main component of the lung rehabilitation exercise plan, but its intervention effect is more significant when performed in combination with other types of exercises [49]. In terms of intensity, the evidence suggests that moderate exercise intensity plays an essential role in health [50]. TCHE is a type of moderate exercise intensity and is considered a safe method of exercise and medical treatment [18, 51]. Furthermore, the use of traditional lung rehabilitation therapy is limited worldwide due to funding, technology, resources, and other conditions [15]. However, TCHEs are more accessible in terms of venue, equipment, cost, and other objective conditions compared with traditional lung rehabilitation therapy [20]. Furthermore, TCHE can function as a means to prevent and treat various chronic diseases, making them a viable alternative to lung rehabilitation therapy.

TCHEs have both direct and indirect impacts on lung functioning. Indirectly, they have a positive effect on lung function by influencing other factors including body mass index (BMI), waist circumference, blood glucose, blood lipid, and blood pressure levels [52]. A previous study found that the TCHEs improve blood pressure [53], BMI [54, 55], waist circumference [55], blood sugar [56], and other variables. Additionally, TCHEs significantly improve depression, anxiety, and sleep quality [57–59]. To explore the different effects of different conditioning methods on the lung function of healthy middle-aged and elderly people, this study distinguishes the different technical movements according to the different conditioning methods. This distinction does not represent a separate relationship between “breath regulation,” “body regulation,” and “heart regulation”; rather, it focuses on the same conditioning methods for integration. Additionally, although these three conditioning methods improve lung function, they differ somewhat in their mechanisms.

This study has some limitations. First, due to the restrictions of the exercise intervention, it is impossible to blind the subjects and coaches, which may cause a certain risk of bias. Because of the nature of the exercise intervention, subjects will know whether they belong to the intervention or control group, which may increase the variability of the intervention in terms of psychological and physiological differences, and in addition may increase the likelihood that the control group will seek to perform the exercise intervention. Additionally, the differences in cultural background and medical environment may also limit the promotion and applicability of TCHEs in other countries and regions. Considering that the intervention could easily be interrupted due to the COVID-19 pandemic, we had to postpone the formal recruitment of subjects. After consulting with relevant experts, we made appropriate modifications and refinements to this protocol.

This paper introduces the design of a clinical trial, which aims to study the effect of different conditioning methods of TCHEs on the lung function of healthy middle-aged and elderly people. Through a comparative analysis of different conditioning methods on the improvement of lung function, this protocol aims to explore the conditioning methods that are more conducive to promoting the improvement of lung function in healthy middle-aged and elderly people and can provide a reference for more targeted exercise prescriptions to improve lung function.

## Trial status

The recruitment of subjects is expected to begin on March 1, 2022, and the study is expected to end on July 1, 2024.

## Supplementary Information

**Additional file 1.** WHO Trial Registration Data Set.

## Data Availability

The datasets used or analyzed in the study can be obtained from the corresponding author upon completion of the study.

## Abbreviations

ANOVA: Analysis of variance
ATS/ERS: American Thoracic Association/European Respiratory Association
BMI: Body mass index
COPD: Chronic obstructive pulmonary disease
FEV1: Forced expiratory volume in 1 s
FVC: Forced vital capacity
MCID: Minimum clinically significant difference
MVV: Maximal voluntary ventilation
RPE: Ratings of perceived exertion
SD: Standard deviation
TCHE: Traditional Chinese health exercise
VC: Vital capacity
