# Supplementary material for: Effect of different conditioning methods of traditional Chinese health exercise on lung function in healthy middle-aged and elderly people: study protocol for a randomized controlled trial
Source: Trials. 2022 Jan 3;23:8. doi: 10.1186/s13063-021-05980-5 (PMC8721470; doi:10.1186/s13063-021-05980-5)
Supplement: Supplementary file 1 — Additional file 1. WHO Trial Registration Data Set. [file 13063_2021_5980_MOESM1_ESM.docx]

| Data Category | Information |
| --- | --- |
| Primary Registry and Trial Identifying Number | China Clinical Trial Registry, ChiCTR2100052687 |
| Date of Registration in Primary Registry | 3 November 2021. |
| Secondary Identifying Numbers | Not applicable |
| Source(s) of Monetary or Material Support | National Social Science Foundation of China |
| Primary Sponsor | National Social Science Foundation of China |
| Secondary Sponsor(s) | Not applicable |
| Contact for Public Queries | Tonggang Fan, fantonggang@126.com |
| Contact for Scientific Queries | Tonggang Fan, Shanghai University of sports, No. 200 Hengren Road, Yangpu District, Shanghai, China； |
| Public Title | Effect of different conditioning methods of traditional Chinese health exercise on lung function of healthy middle-aged and elderly people: A study protocol for a randomized controlled trial |
| Scientific Title | Effect of different conditioning methods of traditional Chinese health exercise  on lung function of healthy middle-aged and elderly people: a randomized controlled trial |
| Countries of Recruitment | China |
| Health Condition(s) or Problem(s) Studied | Health |
| Intervention(s) | traditional Chinese health exercise |
| Key Inclusion and Exclusion Criteria | Inclusion Criteria  (1) aged 45-75 years, male or female;  (2) no history of respiratory diseases;  (3) have not undergone systematic traditional health-preserving exercises in the past, such as Taijiquan and Baduanjin;  (4) fully aware of the research, voluntarily participate, and sign the informed consent form;  Exclusion Criteria  (1) limited physical activity or other factors that hinder the performance of the exercise intervention;  (2) other regular exercise regimens are being performed or will be performed;  (3) cognitive impairment;  (4) participation in other clinical studies. |
| Study Type | Interventional  Allocation: randomized  Intervention model: parallel assignment  Masking: Outcome assessor blinding  Primary purpose: prevention |
| Date of First Enrolment | Expected on 1 March 2022 |
| Sample Size | 480 |
| Recruitment Status | Recruitment has not started |
| Primary Outcome(s) | forced vital capacity (which will be measured  at baseline，3 months, and 6 months) |
| Key Secondary Outcomes | (1) forced expiratory volume in 1 sec  (2) FEV1/FVC  (3) vital capacity  (4) maximal voluntary ventilation  (which will be measured at baseline，3 months, and 6 months) |
| Ethics Review | 1. Status: Approved  2. Date of approval: 1 January 2020  3. Name and contact details of Ethics committee: Ethics Committee for Human Experimental Scientific Research of Shanghai Institute of Physical Education (registration number 102772020RT039), lunli@sus.edu.cn |
| Completion date | 1 July 2024 |
| Summary Results | Not applicable: protocol |
| IPD sharing statement | The datasets used or analysed in the study can be obtained from the corresponding author upon completion of the study. |
